# How could 20-minute neighbourhoods impact health and health inequalities? A policy scoping review

**DOI:** 10.1186/s12889-024-20928-5

**Published:** 2024-12-18

**Authors:** Roxana Pollack, Jonathan R Olsen, Alison Heppenstall, Andreas Hoehn, Jennifer Boyd, Vicki Ponce Hardy, Jennifer Littlejohn, Amy Stevenson, Richard Mitchell, Petra Meier, Jonathan Stokes

**Affiliations:** 1 MRC/CSO Social and Public Health Sciences Unit, School of Health and Wellbeing, College of Medical, Veterinary and Life Sciences, University of Glasgow, Clarice Pears Building, 90 Byres Road, Glasgow, G12 8TB UK; 2 School of Political and Social Sciences, University of Glasgow, Glasgow, UK; 3 Salvation Army Centre for Addiction Services and Research, University of Stirling, Stirling, UK

**Keywords:** 20-minute neighbourhoods, 15-minute cities, Health inequalities, Place-based interventions

## Abstract

**Background:**

‘Twenty-minute neighbourhoods’ (or variations, such as 15-minute cities) are receiving increasing policy attention with anticipated impacts on population health (inequalities) outcomes alongside sustainability improvements. Yet, factors contributing to possible health impacts are not well understood. This scoping review aimed to identify proposed and evidenced pathways to health (inequality) outcomes from international policy plans.

**Methods:**

We first identified relevant academic literature, searching Scopus, (Ovid) Medline and Embase databases. A second search aimed to identify local or national planning or policy documents on government websites and related organisations. We followed a snowball search strategy to retrieve examples identified from the academic literature search and from the C40 cities network. These policy documents were our primary target for extraction, and we extracted and analysed by individual place. Pathways to health and health inequality outcomes identified in these documents were inductively coded thematically. We used Sankey diagrams to visually aggregate the thematic codes for each place relating to pathways to health outcomes and social determinants (mechanisms).

**Results:**

In total, 36 places across 17 countries were included, described across 96 academic articles, policy plans and reports. While different health improvement outcomes were included as a goal in nearly all policy plans, most frequently references were to health in general rather than specific health outcomes. Pathways to health were discussed in numerous policy plans across three overarching themes: proximity, place redesign, and environmental action. Proximity pathways were most frequently outlined as the means to achieve health outcomes, with active travel acting through increased physical activity/reduced obesity as the most frequent individual pathway. However, few plans specified what would actually be implemented in practice to achieve the increased proximity to services. Health inequalities were only mentioned by six places specifically, although nearly half of all places mentioned broader inequality aims (e.g., poverty reduction). Possible unintended consequences to health inequalities also received some attention, for example through displacement of residents.

**Discussion:**

Pathways to assumed health (inequality) outcomes require better specification and evidence. Health inequalities are particularly under-explored, and scenario modelling might provide a means to explore the dynamic aspects necessary to examine these important outcomes pre-implementation.

**Supplementary Information:**

The online version contains supplementary material available at 10.1186/s12889-024-20928-5.

## Background

Liveable, or walkable, neighbourhoods and cities have been conceptualised since the early twentieth century and have increasingly been implemented in recent decades on a global scale [1]. These are based on earlier planning concepts, such as Ebenezer Howard’s 1898 Garden city [2], the ‘compact city’ approach [3], New Urbanism (1980s) and Isobenefit Urbanism (2010s) [4, 5]. Time-based (‘x-minute’) labels are the most recent iteration for these concepts. For instance, the ’20-minute neighbourhood’ has its origins in Portland, Oregon, in the 2010s [6].

These chrono-urbanist concepts share many overlapping characteristics and aims for their implementation, focusing on proximity, diversity and density for greater accessibility and equality of service and space use [7]. Perhaps the most popular version is currently the ’15-minute city’, coined in 2015 at the Paris COP21 conference [8]. This outlines a human-centric, environmentally sustainable concept that allows all residents to access their daily needs – housing, work, food, health, education, culture and leisure – within a 15-minute walk, bike ride or public transport trip [9, 10]. While the specific time/distance (e.g., 15 or 20 minute) and area (e.g., city or neighbourhood) measures differ between policies, the underlying concept of creating walkable places that reduce car dependency runs throughout.

These concepts have gained further attention and policy support by national and local governments in the search for more place-based resilience beyond COVID-19 [11, 12]. For example, the ’15-minute city’ has been planned, adopted, or considered as a strategy to “build back better” as part of the C40 cities network, a collaboration of over 100 cities across many different countries, currently representing one fifth of the global economy [13, 14]. The UK Government’s recent ‘Levelling Up’ plan aims to invest in places with high deprivation and health needs to improve economic and health outcomes for local populations [15]. These policies, already being pitched and trialled in response to climate change and sustainability goals, are now framed as a wider “framework for sustainability, liveability, and health” [16], a ‘multi-solving’ policy approach [9, 10, 14]. For example, the Scottish Government has recently committed to rolling out the ’20-minute neighbourhood’ nationally, and specifically aims to use this as a means to also reduce health inequalities [17], committing initially £325M as part of the Place Based Investment Programme to help kickstart [18, 19].

While there has been a growing body of evidence on the general relationship between health and neighbourhoods, the evidence remains both mixed and unclear on which specific pathways ‘x-minute cities or neighbourhoods’ might implement to improve health while reducing health inequalities [19, 20]. It is essential to explore how different operationalisations of ‘x-minute cities or neighbourhoods’ might aim to impact health and health inequalities to inform these interventions, and their subsequent evaluations, as they are increasingly rolled out (on a global scale).

For this review, we summarised the variety of chrono-urban concepts under the term ‘x-minute cities or neighbourhoods’. To account for its most common characteristics, we have defined ‘x-minute cities or neighbourhoods’ as concepts with the aim of creating spaces where all essential services for daily life are in accessible distance through walking, cycling, or high-quality public transport in residential areas [9, 14]. We scoped academic literature to get to example cases, and then focused on policy documents describing specific planned or implemented approaches to answer our main research question: “How have pathways to health (inequality) outcomes been detailed within the operationalisation descriptions and plans of ‘x-minute cities or neighbourhoods’ and urban areas?”. Our objectives were to:

(i) map the differences in how ‘x-minute cities or neighbourhoods’ concepts are being planned and implemented in practice;
(ii) collate the direct and indirectly outlined path(s) between implementation and (e.g., due to sustainability and modal shifts) positive or negative health (inequality) outcomes.

## Methods

We followed the Arksey & O’Malley (2005) framework for scoping reviews, along with the subsequent methodological enhancements by Levac, Colquhoun & O’Brien and Peters, as well as the PRISMA-ScR Checklist [21–24]. Scoping reviews provide a broad overview of a topic, which is suitable due to the broad range of conceptualisations of ‘x-minute cities or neighbourhoods’ and novelty in implementation. Identifying the research question (Stage 1) is described in detail in the published protocol [25].

### Stages 2 & 3: Identifying and selecting relevant studies

#### Database selection and search strategy

We used a two-stage search strategy to identify both relevant academic literature and grey literature, focused on policy plans and reports. Thus, we first identified practical plans or implementation examples from relevant academic literature, primarily looking for details of the places implementing (or plans) and any details of how they envisioned health (inequality) outcomes to occur. One researcher searched academic literature in the databases Scopus, and the Ovid interface for Medline and Embase separately (11^th^ January 2023). The search included a variety of the conceptual terms used for this type of intervention, determined by C40 cities concepts and input from the research team as well as collaborators with expert knowledge, such as: *-minute city, *-minute neighbo*, Walkable neighbo*, Liveable neighbo*, Compact city, Superblock, Isobenefit urban. A full example search strategy is available in Appendix 1.

For relevant grey literature, we followed a snowball search strategy to retrieve examples identified from the academic literature search (above) and relevant C40 cities [26]. We used this second search to look directly for local or national planning or policy documents on government websites and related organisations. These policy documents were our primary target for extraction since most cities trying to implement these policies are still at a planning stage. Due to our interest in how health outcomes were described on a policy level, this literature was the most relevant for understanding hypothesised mechanisms. However, we also included the relevant academic and other grey literature directly where they presented data relevant to our objectives. The search results were imported into the reference management software Zotero.

#### Study selection

Two phases of screening were conducted, one for academic databases and one for policy document (grey literature) sources. For the academic databases, we first screened titles and abstracts. Three reviewers independently screened two unique 10% random samples iteratively to check for and attend to any discrepancies in screening agreement (measured by Cohen’s kappa) [27]. We used this initial process to set a common understanding of the threshold for clear includes/excludes, and more borderline abstracts among the reviewers. The remaining titles and abstracts were each screened by a single reviewer, with all borderline cases flagged and independently screened by a second reviewer. Two reviewers then independently screened all full texts at the next stage. The final academic articles were used to identify relevant place examples.

For the subsequent policy document search, we combined the places identified from the academic literature screening stage and the C40 city network list [26]. Three reviewers searched for related policy documents independently. The final list of included places was compared again to the inclusion criteria and reached agreement with all reviewers.

The following inclusion criteria were applied, studies that:

1. deal with ‘x-minute neighbourhoods or cities’ (or related concepts with an emphasis on meeting daily needs within a walkable local distance),
2. discuss a concrete example(s) of a planned or realised implementation of these concepts,
3. link to health outcomes, health inequalities or social determinants of health.

Studies were excluded if they were not available in English (language title and abstract) and published prior to 1980.

For the scientific academic literature stage, we concentrated on inclusion criteria 1) and 2) to ensure identification of relevant place examples even if the health aspect was not the focus of the specific scientific article. All final documents identified were screened against the full set of criteria for inclusion.

### Stage 4: Charting the data

Four reviewers individually extracted several papers, checked by an additional reviewer. Data was extracted on bibliographic variables: (article title, author(s), year of publication, journal title), setting variables (study location), concept variables (concept type, implementation status, concept characteristics), health, health inequalities, and social determinants pathways mentioned, and references used to evidence these pathways. We extracted the information without an a priori coding frame for all areas of interest, except for social determinants where we used a World Health Organization framework as guidance [28]. Where health/health inequality/social determinant pathways were supported by references, we also examined the original references to comment on the supporting evidence. The extracted data was compared and discussed to ensure consistency between the researchers. For validation and coding, all data was compiled in the reference management software Rayyan, and analysis and decisions tracked in an Excel spreadsheet.

### Stage 5: Collating, summarising and reporting the results

We first reported the screening results using a PRISMA flow diagram. While we initially planned to extract and compare the concrete policy implementation elements (such as, building a new mixed development, or pedestrianising a street) to answer our first research objective, it became clear that this information was frequently vaguely reported or missing altogether from the documents we extracted. For example, instead of, ‘X, Y, Z are being implemented to achieve a 20-minute neighbourhood…’, the vast majority of policy documents started instead from the policy goal step. For example, ‘achieving a 20-minute neighbourhood will lead to…’, without the details of how that implementation would actually be achieved. For our first research objective, we instead reflected and compared the level and differences of detail in the extracted texts for the individual policy concepts, and whether the document described the policy as already implemented or just planned (in addition to the broad themes, outlined below).

We then summarised the lists of health and health inequality outcomes mentioned in policy plans. For the pathways to health and health inequalities, our main research objective, we inductively coded the extracted information thematically. We created a coding frame through several iterations. These iterations included discussion with the wider team at each stage, contextualising the extracted information and reviewing our background literature on neighbourhood concepts. We continued this process until theoretical saturation was achieved, where we struggled to further distil the categories and we agreed a final consensus.

The final coding frame consisted of three overarching themes with several subthemes of analytical codes for concept elements, mechanisms, and outcomes. Concept elements described the improvements to the built environment and services outlined as components of the policy targets, therefore serving as starting points for pathways. Mechanisms were subsequent hypothesised effects on factors relating to social determinants of health for the target population. Outcomes consisted of proposed effects on health and health inequalities. See Table 1 as an example for coding extracted information.

**Table 1:** Coding of an extract into thematic codes

**Example excerpt (related to proximity)**	**Concept element**	**Mechanism**	**Health outcome**
“Replacing long commute and car journeys with bicycles” -> “reduce vehicle emissions” -> “increase residents health”	Active travel	‘Reduced car emissions/pollution’	Health (general)

Pathways were then conceptualised as having one or more concept elements leading to one or more mechanisms and one or several health (inequalities) outcomes. Pathways were also reported if they only included any two of the components alone, with missing links made explicit. We used this final coding frame and conceptualisation of pathways to compile a Sankey diagram (using R software) of complete and partial pathways to health outcomes and social determinants of health (termed ‘mechanism’). Each place contributing multiple pathways (without allowing an identical duplicated pathway for any single place), which we aggregated visually to show the connections proposed between different parts of the pathways.

For the substantiation of pathways, we extracted direct or noted indirect references in the policy texts if these were cited in relation to a health outcome and/or health pathways. These were then compiled by place and a reviewer followed all references concretely linked to a health outcome and/or pathway to identify their source and content.

## Results

### Search results

**Fig. 1:**
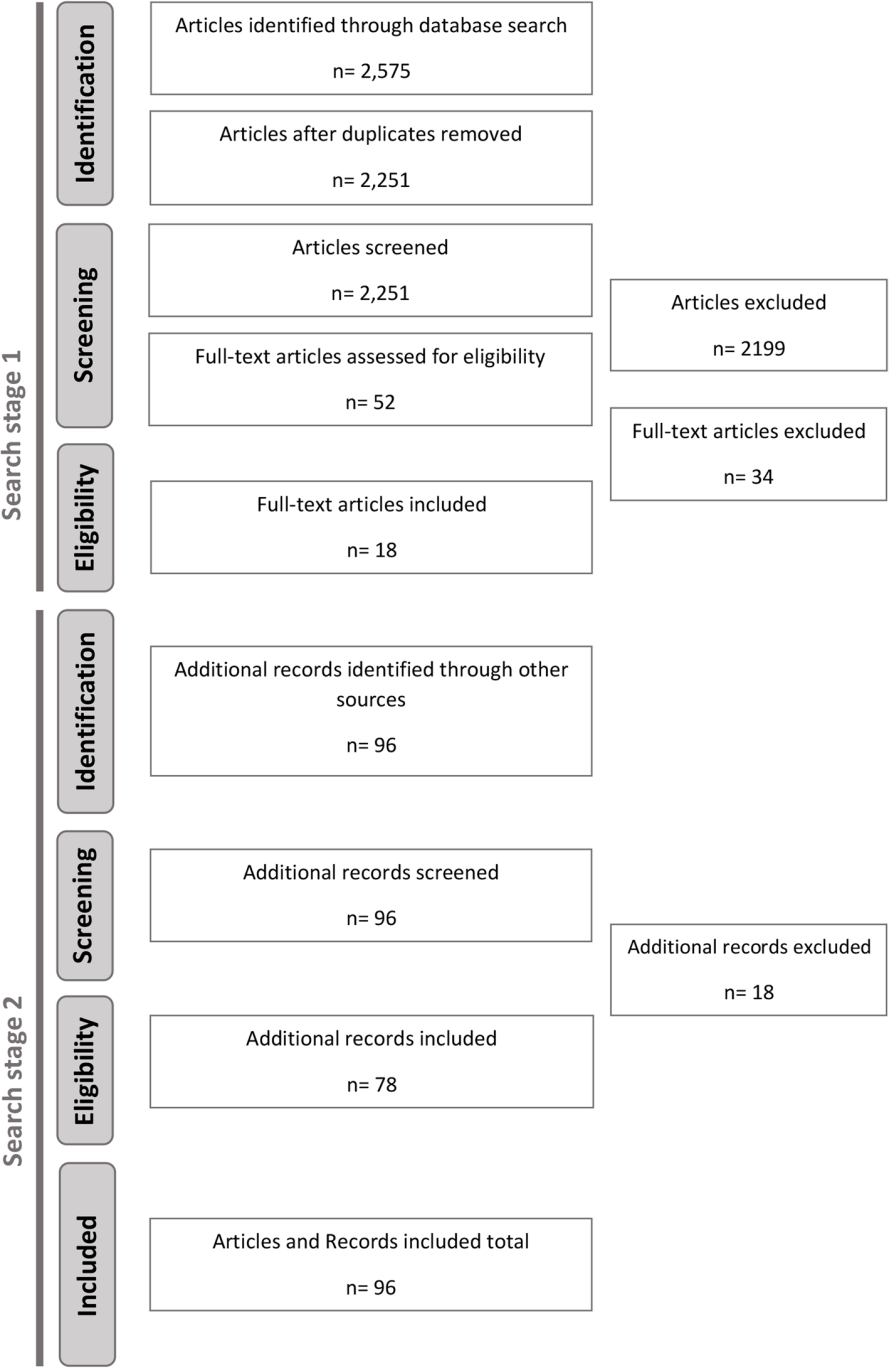
PRISMA flow diagram (*Articles = Academic articles, Records = Public policy plans or documents)

We identified and screened an initial 2,575 studies from the academic literature search (see Figure 1). After screening (search stage 1), we identified 18 academic articles that contained details of places planning or implementing [7, 10, 14, 29–43], which we used for our subsequent grey literature search (combined with the C40 cities list). At the end of the search stage 2, we identified and extracted data from 78 policy records. In total 36 places were included, detailed across these 96 (18+78) academic articles, policy plans and reports.

### Concept characteristics

The 36 places included were from 17 countries (Australia, Spain, Italy, UK, Norway, Portugal, USA, Canada, China, Singapore, New Zealand, Thailand, Hungary, Argentina, Ireland, Denmark, Poland). Within our definition of ‘x-minute cities or neighbourhoods’, we found various policy labels. Most common were different variations of neighbourhood and community-based concepts (e.g. liveable neighbourhoods, walkable communities, 20-minute neighbourhoods), followed by city-wide concepts (e.g. 15-minute city, liveable city). Other more distinct concepts were superblocks and compact cities. In twenty-nine places these policies were planned rather than having any implementation described within the documents (see Table 2).

**Table 2:** Summary of included places

**City, Country**	**Policy labels**	**Stage**	**References**
Perth, Australia^a^	Liveable Neighbourhoods	Implemented	[30, 31, 33–38]
Barcelona, Spain	Superblock	Implemented	[29, 39, 44–46]
Milan, Italy	15-minute city	Implemented	[29, 40, 47, 48]
Hounslow, UK	Low Carbon Neighbourhoods	Planned	[49]
Leeds, UK	20-minute City	Planned	[50]
Newham, UK	15-minute neighbourhood	Planned	[51]
Oslo, Norway	15-minute city concept	Implemented	[52, 53]
Lisbon, Portugal	15-minute city concept	Implemented	[53]
Central Puget Sound Region, USA	Walkable communities	Planned	[54]
Edinburgh, Scotland	20-Minute Neighbourhoods	Planned	[55–58]
Toronto, Canada	complete communities	Planned	[59]
Shanghai, China	15-minute Community Life Circle	Implemented	[43, 60]
Scotland (nation-wide)	20-Minute Neighbourhoods	Planned	[61, 62]
Vancouver, Canada	Complete walkable neighbourhoods	Planned	[63–67]
Singapore, Singapore	20-min towns within 45-min cities	Planned	[68]
Sydney, Australia	20-minute neighbourhood	Planned	[69]
Auckland, New Zealand	Compact urban form	Planned	[70, 71]
Bangkok, Thailand	Compact city	Planned	[72, 73]
Brampton, Canada	20-minute neighbourhood	Planned	[74, 75]
Budapest, Hungary	Compact city/Liveable city	Planned	[76]
Buenos Aires, Argentina	15-minute city	Planned	[77, 78]
Greater Adelaide, Australia	20-minute neighbourhood	Planned	[10, 79–81]
Chicago, USA	Liveable neighbourhoods	Planned	[82–86]
Melbourne, Australia	20-minute city	Planned	[10, 87–93]
Dublin, Ireland	15 min city	Planned	[94–98]
Copenhagen, Denmark	Smart city	Planned	[99–102]
Pleszew, Poland	15-minute city	Implemented	[103]
Portland, USA	Complete neighbourhoods	Planned	[104]
Ottawa, Canada	15-minute city	Planned	[105]
Western Australia^a^	Liveable city	Planned	[106–108]
Hamilton Kirikiriroa, New Zealand	20-minute city	Planned	[109]
Eugene, USA	Walkable neighbourhoods	Planned	[110]
Kirkland, USA	10-minute neighbourhood	Planned	[111–114]
Tempe, USA	20-minute city	Planned	[115]
Greater Bendigo, Australia	10-minute neighbourhood	Planned	[116–119]
Boulder, USA	15-minute neighbourhood	Planned	[120, 121]

^a^We included both Perth and Western Australia separately as Perth was reported separately in many sources (aligned with a specific research project/pilot), which appeared to be slightly different from the broader regional plans

The theme of active travel was the most common characteristic described in the policy plans and consisted of, for instance, reduction in car dependency, active travel infrastructure (cycling, walking), and greater walkability overall. While many of the concepts are defined explicitly by proximity (e.g., the number of minutes to access local services), they also went beyond describing proximity-related changes alone. Second most common were references to service and infrastructure improvements, which included, for example, the improvement and development of public and green infrastructure, service improvement, changes in land use infrastructure, improved housing, density and mixed-use spaces. Characteristics contributing to the improved liveability focused on the creation of more locality and proximity in public spaces, adding more connected and hybrid spaces and bringing community and culture together. Safety-related characteristics concentrated mainly on surveillance and social distancing. Some concepts also outlined aims regarding economic improvement, such as greater focus on and investment in the local economy as well as better work arrangements. Characteristics related to sustainability covered, for instance, creating cleaner environments, reduction in emissions, sustainable mobility, and urban action towards climate justice.

In terms of increasing proximity of services directly, how the concepts were being planned and implemented in practice was mostly vaguely described (mostly with an aim, such as ‘we will create walkable neighbourhoods’, rather than description of how this will be achieved). Where they were described, the concrete interventions broadly fell into three main groupings: 1) bringing services closer to where people live – such as converting ground floors of building, or targeting specific local service locations to existing or new developments; 2) bringing housing (i.e., people) closer to services – again, new developments or redevelopment of existing spaces; 3) reducing the active travel time between housing and services – e.g., via pedestrianisation or cycle paths. Additionally, some interventions sometimes blurred this categorisation slightly, such as an intervention where public school grounds are opened to the public as green space out-of-hours, thereby creating multifunctional urban spaces.

### Inclusion of health outcomes

Thirty-four of the included 36 places had plans which mentioned health outcomes as a possible and targeted outcome of their implementation. It is important to note that outcomes were framed as positive across all policy plans, with little mention of possible negative health outcomes. While health is a common outcome theorised to improve with the implementation of ‘x-minute cities or neighbourhoods’ concepts, it was often considered in broad and unspecific terms. These broader terms for health outcomes can lack clarity on the exact nature of improvements to inhabitants' health. Unspecific descriptions summarised under ‘health’ included, for example, “improved health”, “wellbeing” or “public health”, but also “quality of life”, “healthy communities”, “healthy lifestyles” or even “happiness of life”. More specific descriptions of health included a variety of specific physical and mental health conditions, both non-communicable diseases (‘NCDs’) and more occasionally also ‘communicable diseases’ (e.g., COVID-19). While negative health outcomes were not discussed explicitly, a few places (e.g. Chicago, Auckland, Scotland) discussed the additional need for connected policy intervention on negative health behaviours (e.g. smoking, drug use, gambling, fast food).

### Inclusion of health inequality outcomes

Across the plans, only six places (Newham, Central Puget, Vancouver, Auckland, Hounslow and Chicago) explicitly referenced health inequalities. These focused mainly on initial targeting and a reduction in health disparities of vulnerable groups (e.g., “inequal effects of Covid-19 on ethnic minorities”, “health conditions in vulnerable populations”, “life expectancy gaps in indigenous communities”) or reduction in unequal access to resources or infrastructure (e.g., “access to healthy foods for low-income families”). While ethnic minorities were explicitly mentioned by three places (Newham, Auckland, Chicago), many of the target outcomes focused on other vulnerable populations like elderly people, low-income households, refugees and sex workers. Interestingly, inequality related targets were mentioned more broadly across 14 places, but without linking these explicitly to health (e.g., “inequality”, “poverty”, “social equity”, “gender equality”) or focused on accessibility for specific groups (e.g. improving safe active travel paths for individuals with disabilities, elderly and migrant workers).

### Pathways to health outcomes

Pathways to health outcomes were present in all plans except for three (Perth, Lisbon, Eugene). We found three overarching themes across the starting points for pathways, which matched key characteristics of the policy concepts: proximity, place redesign and environmental action. Proximity relates to policy changes focused on improving the physical access of services and amenities within the area, and change in travel mode to assist greater mobility and accessibility (i.e., bringing people closer to services). Place redesign describes changes related to the quality of services and built infrastructure, related to the broader physical environment or the quality of services themselves. Environmental action consists of environmental and climate change-related changes. We conceptualised pathways as consisting of concept elements related to each theme, mechanisms and health outcomes. While complete pathways would feature all three components, we also included incomplete pathways which aligned with our coding frame. Table 3 outlines the set of thematic codes, and their descriptions generated for the different parts of the pathways. Appendix 2 outlines the pathways by city while grouping their separate health outcomes.

**Table 3:** Summary of health pathway codes

Type	Theme	Short description	Description
Concept element	Proximity	Active travel	Improved public travel or active modes of travel
		Increasing service accessibility	Improved access to services and service-related infrastructure through better transport connectivity and expansion of frequency of services across the urban environment (including access to green and public spaces)
	Place-redesign	Nature access/green or blue infrastructure	Greening city environments through greater quality nature access and/or expanding green or blue spaces
		Improved mobility	Improved quality of transport infrastructure through better quality and connectivity
		Improved built environment	Improved quality of buildings, streets, infrastructure
		Improved service quality	Improved quality of services
		Improved housing	Improved access, affordability and/or quality of housing to meet housing needs
	Environmental action	Net zero	Focus on environmental preservation or climate change prevention/mitigation to achieve carbon neutrality
Mechanisms	Emissions	Reduced car emissions/pollution	Transport related reductions in air pollution, greenhouse gas emissions/environmental emissions
	Health behaviours	Increased physical activity/reduced obesity	Increased daily physical activity through walking and promotion of sport; reduction in obesity
		Accessible/healthy food	Accessible/ affordable/safe fresh food; healthy diet
	Community building	Community liveability	Community building (social cohesion, social interaction, resilient and healthy communities); safety (surveillance, transmission reduction, safe street environments)
	Services	Improved healthcare	Better quality and/or access to healthcare services (e.g., pharmacy or GP)
	Economy	Local economy/employment support	Expansion of job market, improvement of local economy (more shopping options, support for business and innovation)
	Sustainability	Sustainability	Environmental resilience (ecological, biodiversity, natural capital), sustainable infrastructure
Health outcomes		Health (general)	General health terms, such as improved “health”, “wellbeing”, “quality of life”
		Mental health	General mental health terms, such as specifying “mental health” (including stress)
		Physical health	General physical health terms, such as specifying “physical health”
		NCDs	Non-communicable diseases specified explicitly, such as “diabetes”, “asthma”, “chronic disease”
		Accidents	Specified acute incident as an outcome, such as “road traffic accident”
		Communicable disease	Communicable diseases specified explicitly, such as “COVID-19”

#### Proximity-based pathways

**Fig. 2:**
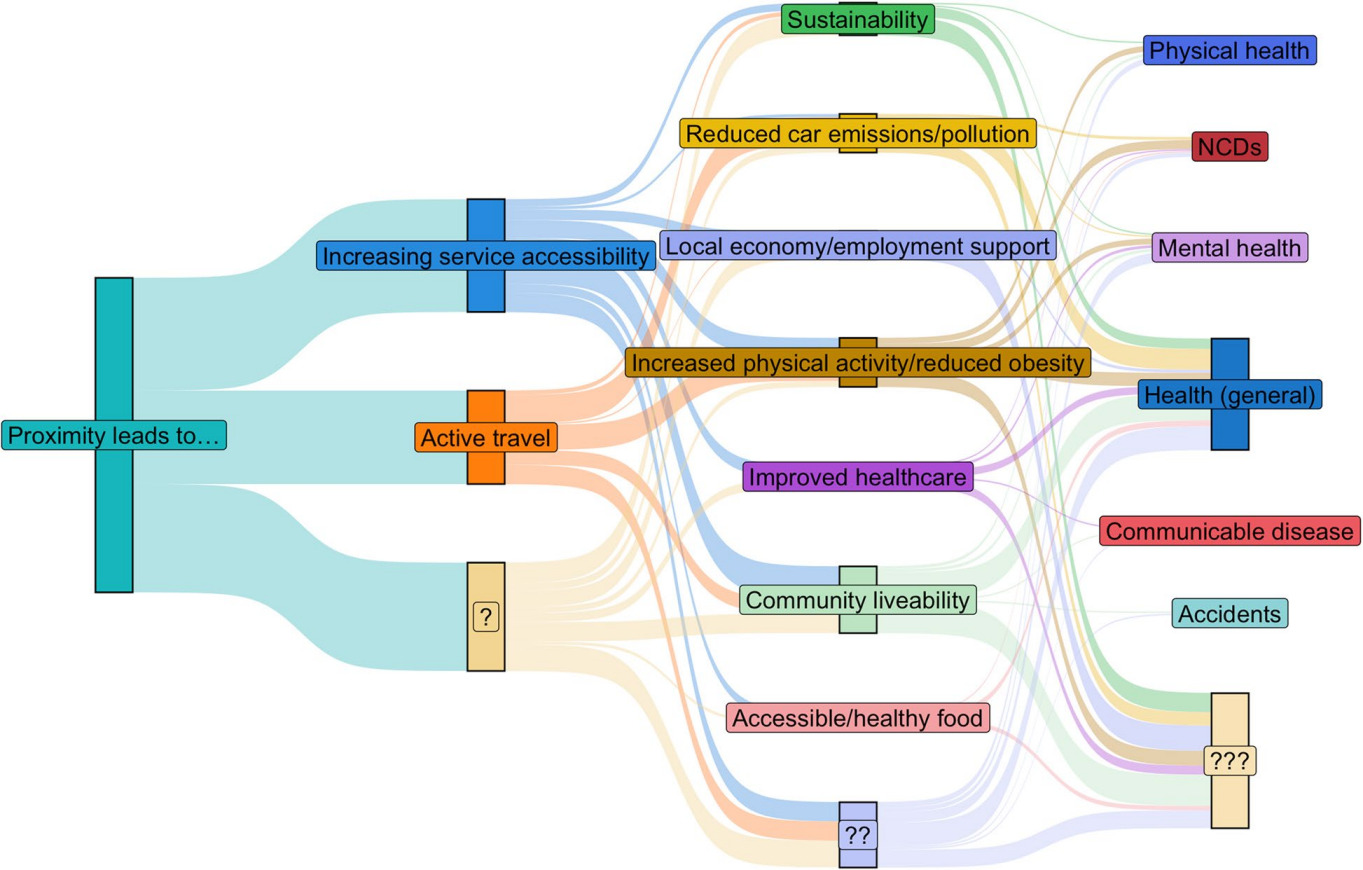
Sankey diagram for proximity pathways (left to right - hypothesised concept elements, mechanisms, and health outcomes). Notes: Question marks indicate where a stage of the pathway was unclear, but other parts of the pathway were outlined (some pathways also finished with a ‘mechanism’ which other places had connected to a health outcome). Size of the bars indicates more places including the connection. Each place could contribute multiple unique pathways, but we de-duplicated within each place. All pathways assume positive benefits at each stage, e.g., decreased non-communicable diseases [NCDs], increased physical health

Proximity-based pathways were the most common and focused on assumed increased active travel and accessibility (/use) of services (see Figure 2). The most common proximity-based pathways were ‘active travel’ leading to ‘increased physical activity/reduced obesity’ and/or ‘reduced car emissions/pollution’. Health outcomes included in these pathways covered a broad range from ‘health’, ‘physical health’, ’NCDs’, to ’mental health’. Pathways starting with ‘increased service accessibility’ most commonly led to ‘community liveability’ and ‘health’, ’mental health’ and ’physical health’. While these concept elements were mainly used in separate pathways, Leeds combined ‘active travel’ and ‘increased service accessibility’ in one of their health pathways.

#### Place redesign pathways

**Fig. 3:**
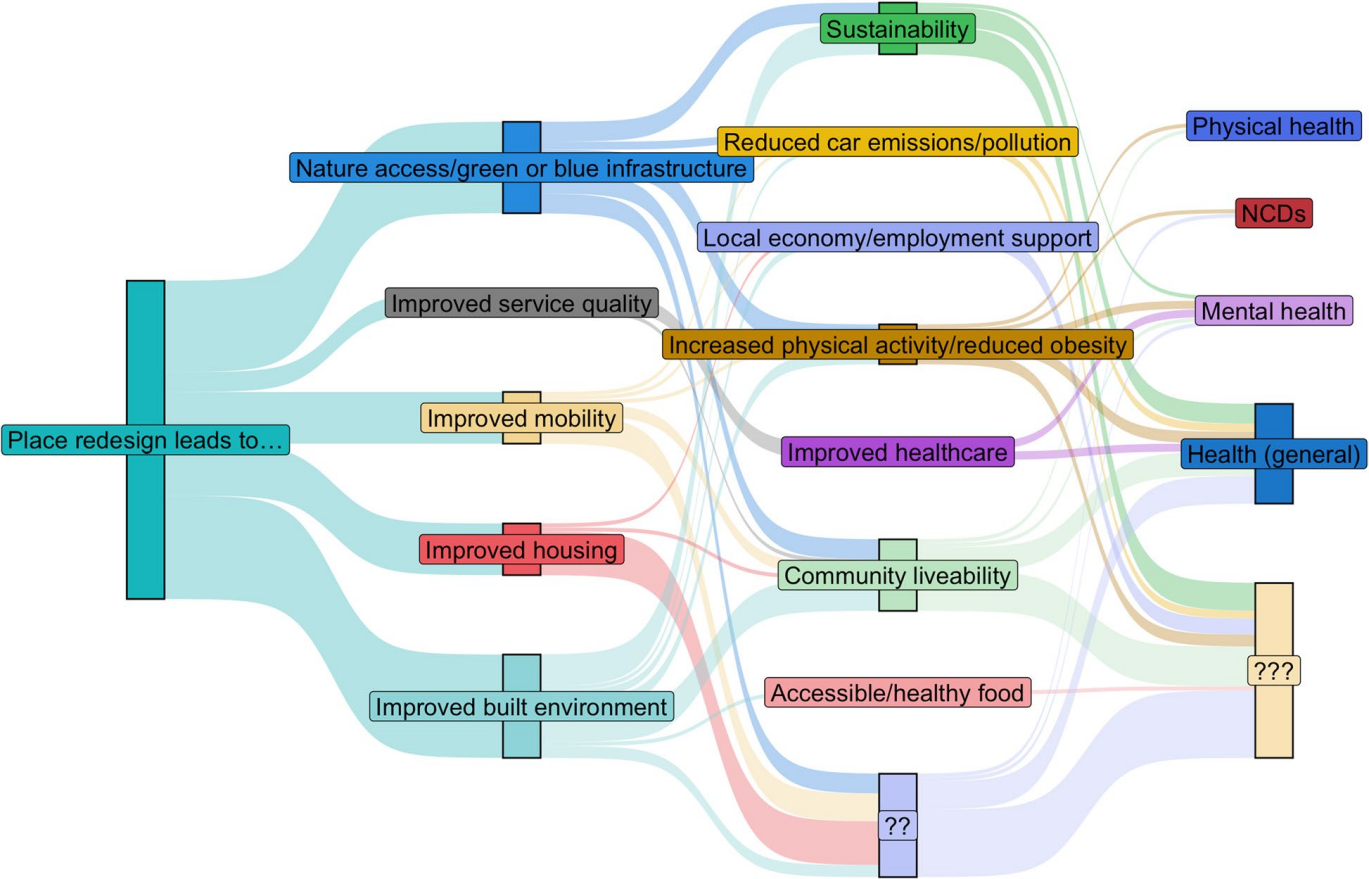
Sankey diagram for place redesign pathways (left to right - hypothesised concept elements, mechanisms, and health outcome benefits). Notes: Question marks indicate where a stage of the pathway was unclear, but other parts of the pathway were outlined (some pathways also finished with a ‘mechanisms’ which other places had connected to a health outcome). Size of the bars indicates more places including the connection. Each place could contribute multiple unique pathways, but we de-duplicated within each place. All pathways assume positive benefits at each stage, e.g., decreased non-communicable diseases [NCDs], increased physical health

Pathways based on place redesign focused on improved quality of services or infrastructure as well as the expansion of public and green spaces (see Figure 3). Paths starting with ‘improved built environment’ were the most common and were proposed to lead to ‘sustainability’ or ‘increased physical activity/reduced obesity’ to health’/’mental health’ outcomes.

Pathways combining concept elements across the themes proximity and place redesign were also common within policy plans. Especially, ‘increased service accessibility’ often paired concept elements from place redesign with ‘community liveability’ as a mechanism. Common examples for these combined pathways were ‘increased service accessibility’ and ‘improved built environment’ leading to ‘community liveability’ and better ‘health, ’mental health’ and ‘physical health’ in three places. ‘Increased service accessibility’ was also combined with ‘nature access/green or blue infrastructure’ by six places, leading to several different mechanisms and improved ’physical health’ and ’mental health’.

#### Environmental action pathways

Pathways based on ‘environmental action’ focused on concept elements related to using strategies to fulfil net zero goals, climate change mitigation and improving environmental conditions. Environmental action was only presumed to lead to health outcomes when combined with one of the other two concept elements (above) and commonly with ‘sustainability’ as a mechanism. While it was mentioned by eleven places, only Barcelona, Greater Adelaide, Sydney included a pathway to health (insufficient for a visualisation).

### Pathways to health inequality outcomes

While health inequalities were included as a target outcome in a few policy plans, they were described without concrete pathways, except for one place plan. Central Puget’s city plan outlined the planned reduction in air pollution leading to a reduced risk in developing related health conditions and improved health of vulnerable populations, such as children, elderly, or chronically ill people. A further 15 place plans (Newham, Milan, Edinburgh, Scotland, Vancouver, Auckland, Bangkok, Brampton, Budapest, Greater Bendigo, Melbourne, Portland, Western Australia, Kirkland, Boulder) included pathways to reduction in inequality and greater equity more broadly (e.g., via reduced poverty), but unconnected to any mentioned health inequalities specifically. Only a few place plans made explicit references to accommodating disabled or elderly people through providing accommodating facilities or modifying footpaths (Bangkok, Western Australia). Common themes in these inequality focused pathways were improved community inclusion, affordable transport and housing, walkable and accessible infrastructure, and better service accessibility. However, importantly, there were also concerns raised in some policy plans about potential for unintended health inequality effects. A few places (Melbourne, Portland, Dublin) briefly drew attention to the potential for increased house prices, gentrification and community displacement, increased local rents and prices potentially forcing out the poorest residents from any benefits accrued.

### Substantiation of pathways

While most places had very few concrete references in their policy plans, eight place plans included concrete references for pathways (Greater Bendigo, Leeds, Newham, Melbourne, Auckland, Singapore, Vancouver, and Edinburgh). These used several types of references to substantiate the pathways described above. For example, government or ministry policy plans (e.g., transport or environmental policy plans), academic research articles, OECD reports, WHO reports, government health department websites, local health information service websites, book chapters, charity reports and even other countries’ ‘x-minute cities or neighbourhoods’ plans. References mainly discussed established links in built environment and transport literature (to wellbeing, sustainability, health), complementary government policy plans, or up-to-date reports on national and international impacts of issues related to urban health. The substantiation of pathways mainly focused on links between changes in transport and subsequent greater walkability to improved physical health (e.g., physical activity, weight loss in children), NCDs, wellbeing as well as sustainability (e.g., better air quality, less pollution). Links were also referenced from changes in transport and built environment to societal benefits, better social connection, reduction in car travel, housing density and economic cost savings.

## Discussion

This scoping review identified 36 places planning or implementing ‘x-minute cities or neighbourhoods’ concepts globally and reviewed their stated potential pathways to health (inequality) outcomes. While the policy concepts included all fulfilled our core definition, they varied in the level of detail on characteristics and effects proposed. A variety of health outcomes were described as target outcomes in nearly all policy plans, but these were mostly generic mentions of ‘health’ and framed as positive outcomes. Negative or unintended health outcomes were very rarely considered. Proximity pathways were most frequently outlined as the means to achieve health outcomes. While ‘active travel’ pathways through reduced car emissions/pollution and physical activity/obesity were the most common specific pathways across policy plans, increased service accessibility was commonly assumed to lead to health effects through community liveability. However, reporting of the plans frequently excluded what would be implemented ‘on the ground’ to achieve the increased proximity to services which, particularly the most recent iterations of the policy, are defined on. Health inequalities were only mentioned by six places specifically, although nearly half of all places mentioned broader inequality aims (e.g., poverty). In addition, possible unintended consequences to determinants of health inequalities received some mention.

### Contextualizing findings within broader literature

Several social determinants identified in this review (included in the pathways to health as mechanisms, e.g., accessible/healthy food, physical activity/obesity) were often framed as beneficial indirect health outcomes themselves. In other words, frequently they were positioned at the end of pathways without any further specification of concrete improvements in defined health outcomes beyond this. Considering the increased emphasis placed on creating walkable neighbourhoods across public health research, this emphasis aligns with the existing literature [122]. However, pathways to direct and indirect health outcomes in policies were often framed in rather deterministic ways, assuming an often linear relationship between urban environments, people’s behaviour, and its effects on health. As a recent paper by Olsen and colleagues has shown, neighbourhoods in Scotland currently fulfilling 20-minute neighbourhood criteria are often some of the most deprived [19]. While changes in travel modes and greater proximity to services can have positive effects on health, there is a greater need for critical engagement with the deterministic and causal assumptions behind some of these pathways (e.g., active travel –> physical activity –> reduced obesity –> reduced heart conditions) within these policies, and whether proximity-based measures alone are a sufficient condition [123, 124].

Since proximity is arguably the key mechanism of ‘x-minute cities or neighbourhoods’ concepts (i.e., the time specified in the title of many), we expected to find a focus on increased active movement throughout the day. In line with this assumption, many pathways to health were conditioned on one or more aspects of improved mobility. However, considerations for individuals or sociodemographic groups who are more likely to have restrictions around movement in achieving this outcome (such as older people or people with disabilities) were barely present in the place plans. This lack of considering diversity in assumptions around what makes places walkable within the target population has already been criticised in reviews of general literature around walkability [124]. While time-based measures of accessibility can be an important predictor of people using services more frequently, these pathways lacked consideration for socioeconomic barriers, physical barriers, or social othering and subsequent variations to the use of services. As pointed out in a recent equity assessment of 20-minute neighbourhoods, an oversight of potential barriers can limit the scope of positive benefits of these measures [125]. Despite the reiteration across several policy plans to create a ‘city for all’, there is a general lack of critical examination of the assumptions behind pathways and their implementation.

The pathway theme centred on place re-design through both better quality and greater accessibility of the built environment and services is in line with other theorised core elements of “healthy neighbourhoods” for creating the potential for positive health effects [122]. A framework on “healthy neighbourhoods”, for example, highlights the importance of public and private service provision, socio-cultural features, and neighbourhood safety [122]. These aspects are included in pathways to health through a focus on community cohesion, cultural engagement, and community safety (through safer street environments or surveillance) as common mechanisms, especially within service accessibility-related pathways. However, changes to the quality of services and built infrastructure often lacked further descriptions on how this change in quality would be achieved. Places that have the key characteristics of ‘x-minute cities or neighbourhoods’ are often cities that were not planned based on motorised travel modes. However, increasing density and land use mix in towns and cities can support this planning concept [126]. The tasks of sufficiently upgrading urban environments in many cases may require quite extensive action and can pose a significant challenge, especially for aims such as affordable and good quality housing [127]. While some place plans referenced investments, it was often left unclear how these changes would be achieved themselves.

Generally, pathways often had gaps and were missing mechanisms or even concept elements relating to the projected policy changes. Plans also mentioned health outcomes without any further specification of pathways to achieve these as clear objectives. This was further ambiguous due to the lack of citing of references to substantiate claims across most places. This lack of connection between theorised benefits of implementing ‘x-minute cities or neighbourhoods’ concepts and concrete ways towards effective implementation to reach these targets was common across different plans. These findings align with previous studies on the lack of clear pathways for evaluation and monitoring of prospective outcomes [10]. Few places (Singapore a notable exception) mentioned potential difficulties with achieving these outcomes due to conflicting demands (e.g. increasing active travel paths accessible to diverse users) and the need to work closely with urban planners (e.g. to effectively reduce road accidents). Clearer operationalised measures for pathways and their evaluation would be especially beneficial when considering the difficulty with implementing integrated policy concepts across the often still siloed departments within local governments.

It is clear from our findings that the inclusion of health inequality as a target outcome in policy plans is least evidenced. Given the discussion of plausible benefits and potential harms to health inequalities within policy plans, this aspect requires significant attention moving forward. A recent cross-sectional examination of socioeconomic differences in associations between living in a 20-minute neighbourhood and diet, physical activity and self-rated health, identified no consistent pattern suggesting living in a 20-minute neighbourhood might not be sufficient to reduce health inequalities [128]. Without consideration of unintended consequences, improved health could result from gentrification of areas with poorer health by wealthier individuals, pricing local populations out and masking ongoing inequalities. As outlined in a recent report by the British Academy and The Academy of Medical Sciences, “understanding geographic inequalities in morbidity and mortality requires…analysis on population movement” [129]. Therefore, careful outlining and examination of possible pathways to improving health inequalities are crucial to create real, long-term, positive impact instead of artificial outcomes exacerbating inequalities.

### Strengths and limitations

To our knowledge, this is the first scoping review to focus on how ‘x-minute cities or neighbourhoods' might impact on health (inequalities) and identify common themes across policies. A strength of this study is the variety of included ‘x-minute cities or neighbourhoods’ policy concepts as search terms, and inclusion of a range of planned or implemented policies provided a broad and international picture of how health outcomes are currently outlined. This further contributes to the current research on the evaluation and monitoring of implementing ‘x-minute cities or neighbourhoods’ concepts. However, there are also several limitations.

The nature of the scoping review method means we are not able to definitively answer whether the health (inequality) outcomes suggested, and pathways to them, are reasonable assumptions. While we also set out to outline how negative or unintended health and health inequalities outcomes were conceptualised, the lack of mentions of possible negative outcomes limited our ability to answer this research objective. Very rarely (if ever) are complex interventions free of unintended consequences, and these would be important to consider and address in future policy planning and evaluation. Additionally, given the early implementation stage of these policies, there is still a lack of medium- or long-term evaluation studies to date. Thus, discussing possible medium- or long-term effects is beyond the scope of this current review, and we focused instead on proposed pathways.

For feasibility, we only included studies in English, and therefore planned or implemented documents for places without an English translation may have been excluded. While we managed to include places from 17 countries, this limits the geographical range of the literature scoped in this review. Due to limitations in the data given in the policy reports, and consistent lack of detail on how health and health inequality outcomes will be achieved in practice, we were not able to extract and illustrate the pathways we identified in the level of detail we initially planned. These documents are often highlighting the policy aspirations for planning rather than the local development plans. We would expect supporting evidence in high level policy plans to outline the proposed benefits of those aspirations. However, we attempted to alleviate this issue by distilling our coding framework down to suitable abstraction to fit the level of data available.

### Implications for policy and research

To achieve the ambitious, and multi-faceted, aims of ‘x-minute cities or neighbourhoods’ concepts, there is likely a need for more coherent logic (or systems) models in policy plans, to help the planning process. This should ideally be combined with more detail of the precise and concrete implementation methods being planned to achieve closer proximity and walkability. Within these assumed models, there is the need to critically examine assumptions and unintended consequences. For example, does having a service close-by necessarily lead to active travel and use of the service, and how do these interact with other factors determining any benefits accrued, such as affordability and quality? This lack of more in-depth consideration of the complexity between people’s behaviour and their surrounding built environment has also been termed “naive environmental determinism”, which can also be applied to the assumed causality within some of the theorised health pathways [127].

Future research could further focus on exploring the changes required to realise the potential benefits of this policy, while avoiding potential harms, and modelling how their implementation can be improved. It might also draw on more recent methodological advances, such as systems science methods and modelling [130]. These methods are better able to identify potential leverage points, possible unintended consequences, and plausible long-term and dynamic impacts – particularly necessary for examining health inequalities in relation to place-based interventions.

### Conclusions

To fulfil their target outcomes, pathways in policy plans should be more detailed, better structured and referenced to aid their effective implementation and subsequent evaluation as multi-solving policies. The complexity of this proposed multi-solving holistic and place-based policy, and the relationships between its components and possible health outcomes, need to be detangled for better and more concrete practical steps towards an effective implementation to reach these goals. Better integration of current evidence and learning from the evaluations of already implemented ‘x-minute cities or neighbourhoods’ concepts is needed to understand and outline more detailed pathways to health and health inequality outcomes.

## Supplementary Information

Supplementary Material 1.

## Data Availability

All data is available from the referenced original sources. The specific data extracts and code for this study are available within an OSF repository (DOI 10.17605/OSF.IO/9AHJD)

## References

[1] Lo RH. Walkability: what is it? J Urban Int Res Placemaking Urban Sustain. 2009;2(2):145–66.

[2] Howard E, Osborn FJ. Garden cities of to-morrow. 11. print. Cambridge: M.I.T. Pr; 2001. p. 168.

[3] Commission of the European Communities. GREEN PAPER ON THE URBAN ENVIRONMENT: COMMUNICATION FROM THE COMMISSION TO THE COUNCIL AND PARLIAMENT. Brussels; 1990 p. 1–63. Available from: https://op.europa.eu/en/publication-detail/-/publication/0e4b169c-91b8-4de0-9fed-ead286a4efb7/language-en. Cited 2024 Apr 15

[4] Congress for the New Urbanism. What is New Urbanism? 2023. Available from: https://www.cnu.org/resources/what-new-urbanism. Cited 2024 Apr 15

[5] D’Acci L. A new type of cities for liveable futures Isobenefit Urbanism morphogenesis. J Environ Manage. 2019;246:128–40. 31176977 10.1016/j.jenvman.2019.05.129

[6] Portland.gov. The Portland Plan. 2024. Available from: https://www.portland.gov/bps/planning/about-bps/portland-plan. Cited 2024 Apr 15

[7] Moreno C, Allam Z, Chabaud D, Gall C, Pratlong F. Introducing the “15-minute city”: Sustainability, resilience and place identity in future post-pandemic cities. Smart Cities. 2021;4(1):93–111.

[8] Moreno C. Carlos Moreno: The 15-minute city. 2020. Available from: https://www.c40knowledgehub.org/s/article/Carlos-Moreno-The-15-minute-city?language=en_US. Cited 2024 Apr 15

[9] O’ Gorman S, Dillon-Robinson R. 20 Minute Neighbourhoods in a Scottish Context. 2021; Available from: https://era.ed.ac.uk/handle/1842/37524. Cited 2022 Oct 11

[10] Thornton LE, Schroers RD, Lamb KE, Daniel M, Ball K, Chaix B, et al. Operationalising the 20-minute neighbourhood. Int J Behav Nutr Phys Act. 2022;19(1):15. 35151334 10.1186/s12966-021-01243-3 PMC8841074

[11] Wittenberg A. CityLab Daily: Is the ‘15-Minute City’ Key to Covid Recovery?. 2020. Available from: https://www.bloomberg.com/news/newsletters/2020-07-16/citylab-daily-is-the-15-minute-city-key-to-covid-recovery. Cited 2024 Apr 15

[12] Sisson P. How the ‘15-Minute City’ Could Help Post-Pandemic Recovery. 2020. Available from: https://www.bloomberg.com/news/articles/2020-07-15/mayors-tout-the-15-minute-city-as-covid-recovery. Cited 2024 Apr 15

[13] C40 Cities. C40 Cities Membership. 2023. Available from: https://www.c40.org/wp-content/uploads/2023/04/C40-Cities-Membership-01_11_23.pdf. Cited 2024 Apr 15

[14] Gower A, Grodach C. Planning innovation or city branding? Exploring how cities operationalise the 20-minute neighbourhood concept. Urban Policy Res. 2022;40(1):36–52.

[15] HM Government. Levelling Up the United Kingdom. London, England; 2022. Available from: https://www.gov.uk/government/publications/levelling-up-the-united-kingdom

[16] Allam Z, Nieuwenhuijsen M, Chabaud D, Moreno C. The 15-minute city offers a new framework for sustainability, liveability, and health. Lancet Planet Health. 2022;6(3):e181-3. 35278381 10.1016/S2542-5196(22)00014-6

[17] Scottish Government. Local Living and 20 Minute Neighbourhoods. Planning Guidance - Draft for Consultation. Edinburgh; 2023 p. 1–79. Available from: https://www.gov.scot/publications/local-living-20-minute-neighbourhoods-planning-guidance/

[18] Our Place. Place Based Investment & Infrastructure. 2024. Available from: https://www.ourplace.scot/about-place/themes/place-based-investment/place-based-investment-infrastructure. Cited 2024 Apr 15

[19] Olsen JR, Thornton L, Tregonning G, Mitchell R. Nationwide equity assessment of the 20-min neighbourhood in the scottish context: a socio-spatial proximity analysis of residential locations. Soc Sci Med. 2022;315:115502. 36368061 10.1016/j.socscimed.2022.115502

[20] McGowan VJ, Buckner S, Mead R, McGill E, Ronzi S, Beyer F, et al. Examining the effectiveness of place-based interventions to improve public health and reduce health inequalities: an umbrella review. BMC Public Health. 2021;21(1):1888. 34666742 10.1186/s12889-021-11852-z PMC8524206

[21] Tricco AC, Lillie E, Zarin W, O’Brien KK, Colquhoun H, Levac D, et al. PRISMA Extension for Scoping Reviews (PRISMA-ScR): Checklist and Explanation. Ann Intern Med. 2018;169(7):467–73. 30178033 10.7326/M18-0850

[22] Arksey H, O’Malley L. Scoping studies: towards a methodological framework. Int J Soc Res Methodol. 2005;8(1):19–32.

[23] Levac D, Colquhoun H, O’Brien KK. Scoping studies: advancing the methodology. Implement Sci. 2010;5(1):69. 20854677 10.1186/1748-5908-5-69 PMC2954944

[24] Peters MDJ, Marnie C, Tricco AC, Pollock D, Munn Z, Alexander L, et al. Updated methodological guidance for the conduct of scoping reviews. JBI Evid Synth. 2020;18(10):2119–26. 33038124 10.11124/JBIES-20-00167

[25] Pollack R, Olsen JR, Heppenstall A, Höhn A, Boyd J, Stevenson A, et al. How could ’x-minute cities or neighbourhoods' impact health? Protocol for a scoping review. figshare. J Contrib. 2023. 10.6084/m9.figshare.21897411.v1.

[26] C40 Cities Climate Leadership Group, C40 Knowledge Hub. 15-minute city initiatives explorer. 2023. Available from: https://www.c40knowledgehub.org/s/article/15-minute-city-initiatives-explorer?language=en_US. Cited 2023 Aug 11

[27] McHugh ML. Interrater reliability: the kappa statistic. Biochem Medica. 2012;22(3):276–82. PMC3900052 23092060

[28] Solar O, Irwin A. A conceptual framework for action on the social determinants of health. Social determinants of health discussion paper 2 (Policy and practice). WHO document production services, Geneva, Switzerland. 2010. p. 1–76. Available from: https://iris.who.int/bitstream/handle/10665/44489/?sequence=1.

[29] Fabris LMF, Camerin F, Semprebon G, Balzarotti RM. New healthy settlements responding to pandemic outbreaks: Approaches from (and for) the global city. Plan J. 2020;5(2):385–406.

[30] Falconer R, Newman P, Giles-Corti B. Is practice aligned with the principles? Implementing New Urbanism in Perth Western Australia. Transp Pol. 2010;17(5):287–94.

[31] Foster S, Hooper P, Knuiman M, Bull F, Giles-Corti B. Are liveable neighbourhoods safer neighbourhoods? Testing the rhetoric on new urbanism and safety from crime in Perth Western Australia. Soc Sci Med. 2016;164:150–7. 25935770 10.1016/j.socscimed.2015.04.013

[32] Gilbert H, Woodcock I. Local Living and Travel Time based Urbanism. Urban Policy Res. 2022; Available from: https://www.scopus.com/inward/record.uri?eid=2-s2.0-85137826410&doi=10.1080%2f08111146.2022.2077327&partnerID=40&md5=3444e03d8a641784dc2906a0e0d9e049

[33] Giles-Corti B, Knuiman M, Pikora TJ, Van Neil K, Timperio A, Bull FC, et al. Can the impact on health of a government policy designed to create more liveable neighbourhoods be evaluated? An overview of the RESIDential Environment Project. New South Wales Public Health Bull. 2007;18(11–12):238–42. 10.1071/nb07027 18093466

[34] Giles-Corti B, Knuiman M, Timperio A, Van Niel K, Pikora TJ, Bull FCL, et al. Evaluation of the implementation of a state government community design policy aimed at increasing local walking: Design issues and baseline results from RESIDE Perth Western Australia. Prev Med. 2008;46(1):46–54. 17881044 10.1016/j.ypmed.2007.08.002

[35] Hooper P, Foster S, Bull F, Knuiman M, Christian H, Timperio A, et al. Living liveable? RESIDE’s evaluation of the “Liveable Neighborhoods” planning policy on the health supportive behaviors and wellbeing of residents in Perth, Western Australia. SSM - Popul Health. 2020;10. Available from: https://www.scopus.com/inward/record.uri?eid=2-s2.0-85079074779&doi=10.1016%2fj.ssmph.2020.100538&partnerID=40&md5=6ef5e442ee83cec8279702158a69b257 10.1016/j.ssmph.2020.100538 PMC7016024 32072006

[36] Hooper P, Giles-Corti B, Knuiman M. Evaluating the implementation and active living impacts of a state government planning policy designed to create walkable neighborhoods in Perth Western Australia. Am J Health Promot. 2014;28(SUPPL 3):S5-18. 24380466 10.4278/ajhp.130503-QUAN-226

[37] Hooper P, Knuiman M, Bull F, Jones E, Giles-Corti B. Are we developing walkable suburbs through urban planning policy? Identifying the mix of design requirements to optimise walking outcomes from the “Liveable Neighbourhoods” planning policy in Perth, Western Australia. Int J Behav Nutr Phys Act. 2015;12(1). Available from: https://www.scopus.com/inward/record.uri?eid=2-s2.0-84930635481&doi=10.1186%2fs12966-015-0225-1&partnerID=40&md5=61ac3431292cd8601ae05a3e8b958b51 10.1186/s12966-015-0225-1 PMC4438522 25981916

[38] Hooper P, Knuiman M, Foster S, Giles-Corti B. The building blocks of a “Liveable Neighbourhood”: Identifying the key performance indicators for walking of an operational planning policy in Perth Western Australia. Health Place. 2015;36:173–83. 26606456 10.1016/j.healthplace.2015.10.005

[39] López I, Ortega J, Pardo M. Mobility infrastructures in cities and climate change: An analysis through the superblocks in Barcelona. Atmosphere. 2020;11(4). Available from: https://www.scopus.com/inward/record.uri?eid=2-s2.0-85085143572&doi=10.3390%2fATMOS11040410&partnerID=40&md5=ea4a43b8d570f8904a5b67942b9acab4

[40] Pinto F, Akhavan M. Scenarios for a Post-Pandemic City: urban planning strategies and challenges of making “Milan 15-minutes city.” Transp Res Procedia. 2022;60:370–7.

[41] Ramirez Saiz A., Jimenez Martin D., Lamiquiz P., Alonso A. The Level of Inclusiveness of Current 15-Minute City Models. A Qualitative Analysis on How Far City of Proximity Strategies and Design for All Are Merging. Stud Health Technol Inform. 2022;297((Ramirez Saiz, Lamiquiz, Alonso) Polytechnical University of Madrid):288–95. 10.3233/SHTI220851 36073406

[42] Rueda S. Superblocks for the design of new cities and renovation of existing ones: Barcelona’s case. In: Integrating Human Health into Urban and Transport Planning: A Framework. 2018. p. 135–53. Available from: https://www.scopus.com/inward/record.uri?eid=2-s2.0-85063119264&doi=10.1007%2f978-3-319-74983-9_8&partnerID=40&md5=a49cb2c86ca1dfab88cd444a8091e22f

[43] Wu H, Wang L, Zhang Z, Gao J. Analysis and optimization of 15-minute community life circle based on supply and demand matching: A case study of Shanghai. PLoS ONE. 2021;16(8 August). Available from: https://www.scopus.com/inward/record.uri?eid=2-s2.0-85114288075&doi=10.1371%2fjournal.pone.0256904&partnerID=40&md5=3dbe2d804c0a21b9b06ad0b3ad8945c7 10.1371/journal.pone.0256904 PMC8407582 34464423

[44] Ajuntament de Barcelona. Barcelona green infrastructure and biodiversity plan 2020. 2013. Available from: https://ajuntament.barcelona.cat/ecologiaurbana/sites/default/files/Barcelona%20green%20infrastructure%20and%20biodiversity%20plan%202020.pdf. Cited 2024 Apr 15

[45] Ajuntament de Barcelona. LET’S FILL STREETS WITH LIFE Establishing Superblocks in Barcelona. 2016. Available from: https://ajuntament.barcelona.cat/ecologiaurbana/sites/default/files/en_gb_MESURA%20GOVERN%20SUPERILLES.pdf. Cited 2024 Apr 16

[46] Nieuwenhuijsen M, Khreis H, editors. Integrating Human Health into Urban and Transport Planning: A Framework. Cham: Springer International Publishing; 2019. Available from: http://link.springer.com/10.1007/978-3-319-74983-9. Cited 2024 Apr 16

[47] Comune de Milano. Milan 2020 Adaptation strategy Open document to the city’s contribution. Available from: https://www.comune.milano.it/documents/20126/7117896/Milano+2020.+Adaptation+strategy.pdf/d11a0983-6ce5-5385-d173-efcc28b45413?t=1589366192908. Cited 2024 Apr 16

[48] Comune de Milano. Milan 2020. Adaptation strategy. Available from: https://www.comune.milano.it/documents/20126/7117896/Open+streets.pdf/d9be0547-1eb0-5abf-410b-a8ca97945136?t=1589195741171. Cited 2024 Apr 16

[49] Cranford and Heston. LONDON BOROUGH OF HOUNSLOW FUTURE NEIGHBOURHOODS 2030. Available from: https://www.hounslow.gov.uk/downloads/file/3803/future_neighbourhoods_2030. Cited 2024 Apr 16

[50] MacDonald M. Leeds 20-Minute Neighbourhoods Technical Note. 2022. Available from: https://www.leeds.gov.uk/docs/Local%20Plan%20Update/Local%20Plan%20Update%20-%2020%20Minute%20Neighbourhoods%20Report.pdf. Cited 2024 Apr 16

[51] Newham London. 15 MINUTE NEIGHBOURHOODS DELIVERY PLAN. Available from: https://www.newham.gov.uk/downloads/file/3925/newham-15-min-neighbourhoods-appendix-1-delivery-plan-redacted. Cited 2024 Apr 16

[52] The Ministry of Local Government and Modernisation. NETWORK OF PUBLIC SPACES IDEAS STRATEGIES EXAMPLES – AN IDEA HANDBOOK. 2019. Available from: https://www.regjeringen.no/contentassets/c6fc38d76d374e77ae5b1d8dcdbbd92a/kmd_public-spaces_innmat_eng_org.pdf. Cited 2024 Apr 16

[53] Di Marino M, Tomaz E, Henriques C, Chavoshi SH. The 15-minute city concept and new working spaces: a planning perspective from Oslo and Lisbon. Eur Plan Stud. 2023;31(3):598–620.

[54] Puget Sound Regional Council. VISION 2040. 2009. Available from: https://www.psrc.org/sites/default/files/2022-07/v2040.pdf. Cited 2024 Apr 16

[55] Policy and Sustainability Committee. 20-Minute Neighbourhood Strategy: Living Well Locally. 2021. Available from: https://democracy.edinburgh.gov.uk/documents/s34667/Item%207.10%20-%2020-Minute%20Neighbourhood%20Strategy%20-%20Living%20Well%20Locally.pdf. Cited 2024 Apr 16

[56] The City of Edinburgh Council. CITY MOBILITY PLAN 2021-2030 Implementation Plan. Available from: https://www.edinburgh.gov.uk/downloads/file/29776/the-city-mobility-implementation-plan Cited 2024 Apr 16

[57] The City of Edinburgh Council. CITY MOBILITY PLAN 2021-2030. Available from: https://www.edinburgh.gov.uk/downloads/file/29320/city-mobility-plan-2021-2030. Cited 2024 Apr 16

[58] Edinburgh Poverty Commission. A Just Capital Actions to End Poverty in Edinburgh. 2020. Available from: https://edinburghpovertycommission.org.uk/wp-content/uploads/2020/09/20200930_EPC_FinalReport_AJustCapital.pdf. Cited 2024 Apr 16

[59] City of Toronto. PROPOSED REDEVELOPMENT OF THE DOWNSVIEW LANDS. 2021. Available from: https://www.id8downsview.ca/_files/ugd/4ea6e4_83b2b0b8db67436782994a36ab9e5c3a.pdf. Cited 2024 Apr 16

[60] Shanghai Urban Planning and Land Resource Administration Bureau. Shanghai Master Plan 2017-2035. 2018. Available from: https://www.shanghai.gov.cn/newshanghai/xxgkfj/2035004.pdf. Cited 2024 Apr 16

[61] Scottish Government. Protecting Scotland, Renewing Scotland: The Government’s programme for Scotland 2020-2021. Edinburgh; 2020 p. 139. Available from: Cited 2022 Oct 18https://www.gov.scot/publications/protecting-scotland-renewing-scotland-governments-programme-scotland-2020-2021/

[62] Scottish Government. National Planning Framework 4. Edinburgh; 2023 p. 1–161. Available from: https://www.gov.scot/binaries/content/documents/govscot/publications/strategy-plan/2023/02/national-planning-framework-4/documents/national-planning-framework-4-revised-draft/national-planning-framework-4-revised-draft/govscot%3Adocument/national-planning-framework-4.pdf. Cited 2023 Aug 11

[63] City of Vancouver. Climate Emergency Annual Report 2022 Indicator and Financial Dashboard. 2023. Available from: https://vancouver.ca/files/cov/ceap-2022-annual-report.pdf. Cited 2024 Apr 18

[64] City of Vancouver. Climate Emergency Action Plan Summary 2020-2025. 2020. Available from: https://vancouver.ca/files/cov/climate-emergency-action-plan-summary.pdf. Cited 2024 Apr 18

[65] City of Vancouver. Greenest City 2020 Action Plan Part Two: 2015-2020. 2015. Available from: https://vancouver.ca/files/cov/greenest-city-2020-action-plan-2015-2020.pdf. Cited 2024 Apr 18

[66] City of Vancouver. Renewable City Action Plan. 2018. Available from: https://vancouver.ca/files/cov/renewable-city-action-plan-summary.pdf. Cited 2024 Apr 18

[67] General manager of planning, Urban design and sustainability and general manager of engineering services. Climate emergency action plan. RECOMMENDATIONS FOR HOW WE MOVE Report. 2020. Available from: https://council.vancouver.ca/20201103/documents/p1.pdf. Cited 2024 Apr 18.

[68] Land Transport Authority. Land Transport Masterplan 2040. Available from: https://www.lta.gov.sg/content/dam/ltagov/who_we_are/our_work/land_transport_master_plan_2040/pdf/LTA%20LTMP%202040%20eReport.pdf. Cited 2024 Apr 18

[69] GREATER SYDNEY REGION PLAN, Greater Sydney Commission. GREATER SYDNEY REGION PLAN A Metropolis of Three Cities – connecting people. 2018 p. 1–194.

[70] Minister of Local Government. The Auckland Plan The World’s Most Liveable City. Available from: https://www.aucklandcouncil.govt.nz/plans-projects-policies-reports-bylaws/our-plans-strategies/Documents/auckland-plan-2012-full-document.pdf. Cited 2024 Apr 18

[71] Auckland Council. Auckland Plan 2050. 2018. Available from: https://www.aucklandcouncil.govt.nz/plans-projects-policies-reports-bylaws/our-plans-strategies/auckland-plan/about-the-auckland-plan/docsprintdocuments/auckland-plan-2050-print-document.pdf. Cited 2024 Apr 18

[72] Strategy and Evaluation Department Bangkok Metropolitan Administration, Faculty of Political Sciences, Chulalongkorn University. Executive Summary 20-year Development Plan for Bangkok Metropolis. Available from: https://webportal.bangkok.go.th/public/user_files_editor/130/BMA-developmentplan/P20ys(2556-2575)sumEN.pdf. Cited 2024 Apr 18

[73] Strategy and Evaluation Department, Bangkok Metropolitan Administration, Faculty of Political Sciences, Chulalongkorn University. EXECUTIVE SUMMARY 20-year Development Plan for Bangkok Metropolis Phase 1 (2013-2017). Cited 2024 Apr 18https://webportal.bangkok.go.th/public/user_files_editor/130/BMA-developmentplan/P20ys_phase1(2556-2560)sumEN.pdf

[74] Beasley and Associates, Planning Inc. LIVING THE MOSAIC Brampton 2040 Vision. 2018. Available from: https://www1.brampton.ca/EN/City-Hall/Brampton-2040-Vision/Documents/brampton2040Vision.pdf. Cited 2024 Apr 18

[75] ULI Toronto. Implementing “20-min Walkable Neighbourhood with Community Hub” as the New Growth Model for Transit-Oriented Communities. Available from: https://ulidigitalmarketing.blob.core.windows.net/ulidcnc/sites/14/2020/10/Uptown-Brampton-Transit-Oriented-Communities-Toolkit-FINAL.pdf. Cited 2024 Apr 18

[76] Urban Development Department of the Municipality of Budapest Mayor’s Office. Budapest 2030 Long-term Urban Development Concept. 2014. Available from: https://archiv.budapest.hu/sites/english/Documents/Urban%20Development%20Plans/Budapest2030_ENG_summary.pdf. Cited 2024 Apr 18

[77] Buenos Aires Ciudad. Second Action Plan of the City of Buenos Aires. Available from: https://www.opengovpartnership.org/wp-content/uploads/2018/12/Buenos-Aires_Action-Plan_2018-2020_EN.pdf. Cited 2024 Apr 18

[78] Buenos Aires Ciudad. CITIES ON A HUMAN SCALE IN THE COVID-19 CONTEXT. 2020. Available from: https://buenosaires.gob.ar/climateaction/post-covid-19-recovery. Cited 2024 Apr 18.

[79] Government of South Australia. 30-Year Plan for Greater Adelaide – 2017 Update Report Card. 2017. Available from: https://livingadelaide.sa.gov.au/__data/assets/pdf_file/0011/893927/30-Year_Plan_for_Greater_Adelaide_-_2017_Update_Report_Card_-_2020-21.pdf. Cited 2024 Apr 18

[80] Government of South Australia. PEOPLE AND NEIGHBOURHOODS POLICY DISCUSSION PAPER. 2019. Available from: https://plan.sa.gov.au/__data/assets/pdf_file/0011/584993/People_and_Neighbourhoods_Policy_Discussion_Paper.pdf. Cited 2024 Apr 18

[81] Government of South Australia. THE 30-YEAR PLAN FOR GREATER ADELAIDE. 2017. Available from: https://livingadelaide.sa.gov.au/__data/assets/pdf_file/0003/319809/The_30-Year_Plan_for_Greater_Adelaide.pdf. Cited 2024 Apr 18

[82] Chicago Mayor Lori E. Lightfoot. WE WILL CHICAGO A framework plan for the city’s future DRAFT FOR PUBLIC INPUT. 2022. Available from https://www.chicago.gov/city/en/sites/dpd-plan-archive/home/we-will-chicago.html. Cited 2024 Apr 18.

[83] City of Chicago. OPEN SPACE. Available from: https://www.chicago.gov/content/dam/city/depts/zlup/Sustainable_Development/Publications/Green%20Healthy%20Neighborhoods/GreenHealthyNeighborhoods_PC_Low_Res_pt_3.pdf. Cited 2024 Apr 18

[84] ULI Chicago. LASALLE STREET Building a Thriving Future Chicago - IL. 2022. Available from: https://ulidigitalmarketing.blob.core.windows.net/ulidcnc/sites/10/2022/06/LaSalleTAP_FinalReport.pdf. Cited 2024 Apr 18

[85] The City of Chicago. The Chicago Central Area Plan Preparing the Central City for the 21st Century Draft Final Report to the Chicago Plan Commission. 2003. Available from: https://www.chicago.gov/content/dam/city/depts/zlup/Planning_and_Policy/Publications/Central_Area_Plan_DRAFT/01_Central_Area_Plan_Intro.pdf. Cited 2024 Apr 18

[86] The City of Chicago. Chicago Central Area Plan Chapter 4 : THEME 1 Development Framework. 2003. Available from: https://www.chicago.gov/content/dam/city/depts/zlup/Planning_and_Policy/Publications/Central_Area_Plan_DRAFT/06_Central_Area_Plan_Chapter4_1.pdf. Cited 2024 Apr 18

[87] Victoria State Government. FIVE-YEAR IMPLEMENTATION PLAN PLAN MELBOURNE 2017-2050. 2017. Available from: https://railfreightalliance.com/wp-content/uploads/2014/06/Plan_Melbourne_2017_Implementation_plan.pdf. Cited 2024 Apr 18

[88] Angelopoulos S, Boymal J, de Silva A. Identifying and valuing the economic benefits of 20-minute neighbourhoods Higher density mixed use and walkability dimensions. 2019. Available from: https://www.planning.vic.gov.au/__data/assets/pdf_file/0025/653254/Economic-benefits-of-20-minute-neighbourhoods.pdf. Cited 2024 Apr 18

[89] The State of Victoria Department of Environment, Land, Water and Planning. Plan Melbourne 2017 - 2050 Addendum 2019. 2019. Available from: https://www.planning.vic.gov.au/__data/assets/pdf_file/0027/628650/plan-melbourne-addendum-2019.pdf. Cited 2024 Apr 18

[90] Victoria State Government. PLAN MELBOURNE 2017-2050 A Global City of Opportunity and Choice Summary. 2017. Available from: https://www.planning.vic.gov.au/__data/assets/pdf_file/0025/628234/plan-melbourne-2017-2050-summary.pdf. Cited 2024 Apr 18

[91] Victoria State Government. METROPOLITAN PLANNING STRATEGY PLAN MELBOURNE 2017-2050. 2017. Available from: https://www.planning.vic.gov.au/__data/assets/pdf_file/0025/654550/Plan_Melbourne_2017-2050_Strategy_.pdf. Cited 2024 Apr 18

[92] Victoria State Government. PLAN MELBOURNE 2017-2050 Report on Progress 2019. 2019. Available from: https://apo.org.au/sites/default/files/resource-files/2020-03/apo-nid302942.pdf

[93] Victoria State Government. 20-minute neighbourhoods - Creating a more liveable Melbourne. 2019. Available from: https://www.planning.vic.gov.au/guides-and-resources/strategies-and-initiatives/20-minute-neighbourhoods/20-minute-neighbourhood-projects. Cited 2024 Apr 18

[94] Dublin City Council. 15 Minute City Concept Planning SPC. 2022. Available from: https://councilmeetings.dublincity.ie/mgConvert2PDF.aspx?ID=35933. Cited 2024 Apr 20

[95] Dublin Chamber. DUBLIN: THE 15 MINUTE CITY. Available from: https://bartra.ie/wp-content/uploads/2020/10/Dublin_The-15-Minute-City.pdf. Cited 2024 Apr 20

[96] Dublin City Council. Dublin City Development Plan 2022-2028 | Executive Summary. 2022. Available from: https://www.dublincity.ie/sites/default/files/2022-12/Final%201-00%20Executive%20Summary.pdf. Cited 2024 Apr 20

[97] Dublin City Council. Dublin City Development Plan 2022-2028 Chapter 5: Quality Housing and Sustainable Neighbourhoods. 2022. Available from: https://www.dublincity.ie/sites/default/files/2022-12/Final%201-05%20Quality%20Housing.pdf. Cited 2024 Apr 20

[98] Dublin Chamber. Renewing Our City Planning for Our Future. Available from: https://www.dublinchamber.ie/Portals/0/Submissions/Dublin-Chamber-Renewing-Our-City-Planning-for-our-Future.pdf?ver=2021-09-22-140843-813. Cited 2024 Apr 20

[99] Quélin and Smadja. THE GREEN GROWTH CITY Copenhagen. 2021. Available from: https://www.hec.edu/sites/default/files/documents/Copenhagen-Smartcities-the-sustainable-program-six-leading-cities-soreport-2021-2%5B4%5D.pdf. Cited 2024 Apr 20

[100] Danish Ministry of the Environment. The Finger Plan A Strategy for the Development of the Greater Copenhagen Area. 2015. Available from: https://www.observatorio2030.com/sites/default/files/2019-11/BP_98_1947_DK_26_The%20Finger%20Plan.pdf. Cited 2024 Apr 20

[101] Hjollund T, Boldt J, Hendriksen N, Sieverts P. Copenhage, Nordhavn Implementation Plan. 2014. Available from: http://www.transformyourcity.eu/resources/transmethod06/IP_Copenhagen.pdf. Cited 2024 Apr 20

[102] The City of Copenhagen Department of Finance. THE CAPITAL OF SUSTAINABLE DEVELOPMENT THE CITY OF COPENHAGEN’S ACTION PLAN FOR THE SUBSTAINABLE DEVELOPMENT GOALS. Available from: https://international.kk.dk/sites/default/files/2022-01/Verdensmål_UK_WEB_FIN.pdf. Cited 2024 Apr 20

[103] Ptak A. Compact Pleszew A 15-minute town. Available from: https://miasto15.pl/wp-content/uploads/2021/07/01-album-MINI-kompaktowy-pleszew-ang.pdf. Cited 2024 Apr 20

[104] Portland Plan Partners. The Portland Plan. 2012. Available from: https://www.portland.gov/bps/planning/documents/portland-plan/download. Cited 2024 Apr 20

[105] City of Ottawa Planning, Infrastructure and Economic Development. 15-MINUTE NEIGHBOURHOODS BASELINE REPORT. 2021. Available from: https://engage.ottawa.ca/8204/widgets/36458/documents/66497. Cited 2024 Apr 20

[106] Western Australian Planning Commission, Department for Planning and Infrastructure. Liveable Neighbourhoods a Western Australian Government sustainable cities initiative. 2009. Available from: https://www.wa.gov.au/system/files/2021-05/FUT_LN_Liveable_Neighbourhoods_update_02.pdf. Cited 2024 Apr 20

[107] Department of Planning, Western Australian Planning Commission. Liveable Neighbourhoods Draft 2015. 2015. Available from: https://www.wa.gov.au/system/files/2021-05/FUT-LiveableNeighbourhoods_2015.pdf. Cited 2024 Apr 20

[108] Department of Planning, Western Australian Planning Commission. Liveable Neighbourhoods Background Information Review of Liveable Neighbourhoods. 2015. Available from: https://www.wa.gov.au/system/files/2021-05/FUT-LN_background.pdf. Cited 2024 Apr 20

[109] Hamilton City Council. Our vision for Hamilton Kirikiriroa. 2020. Available from: https://indd.adobe.com/view/6127dbd0-1a87-4fad-96ae-fefd42775c88. Cited 2024 Apr 20

[110] envision Eugene. Envision Eugene Comprehensive Plan. 2017. Available from: https://www.eugene-or.gov/DocumentCenter/View/71354/Envision-Eugene-Comp-Plan---August-2023. Cited 2024 Apr 20

[111] City of Kirkland. CITY OF KIRKLAND SUSTAINABILITY MASTER PLAN. 2020. Available from: https://www.kirklandwa.gov/files/sharedassets/public/v/1/public-works/recycling/sustainability/sustainability-master-plan-adopted-dec-2020.pdf. Cited 2024 Apr 20

[112] City of Kirkland. City of Kirkland Transportation Master Plan. 2015. Available from: https://www.kirklandwa.gov/files/sharedassets/public/v/2/public-works/city-of-kirkland-transportation-master-plan.pdf. Cited 2024 Apr 20

[113] Planning Commission. 2044 Comprehensive Plan Update. 2023. Available from: https://www.kirklandwa.gov/files/sharedassets/public/v/1/planning-amp-building/kirkland-2044-comp-plan/pc-presentation-briefing-status-update-vision-statement-policy-changes-07272023.pdf. Cited 2024 Apr 20

[114] City of Kirkland. Parks, Recreation & Open Space Plan. 2022. Available from: https://www.kirklandwa.gov/files/sharedassets/public/v/1/parks-amp-comm-services/park-planning/pdfs/2022-pros-plan-from-memo.pdf

[115] Community Development Department. City of Tempe General Plan 2040. 2020. Available from: https://www.tempe.gov/home/showpublisheddocument/86155/637395866769170000

[116] Compact Greater Bendigo. GREATER BENDIGO PLANNING SCHEME. Available from: https://cumberland-files.s3.amazonaws.com/a18a00ee4d1dff89e4c346021e5c8469.pdf. Cited 2024 Apr 20

[117] City of Greater Bendigo. Connecting Greater Bendigo Integrated Transport and Land Use Strategy. 2015. Available from: https://www.bendigo.vic.gov.au/sites/default/files/2023-10/City-Greater-Bendigo-Integrated-Transport-and-Land-Use-Strategy-2015.pdf. Cited 2024 Apr 20

[118] City of Greater Bendigo. Greater Bendigo Residential Strategy Vol. 2 – Analysis, Strategy & Implementation. 2014 p. 1–85.

[119] City of Greater Bendigo. Greater Bendigo Residential Strategy Adopted Version v.03. 2016 p. 1–14.

[120] City of Boulder, Boulder County. BOULDER VALLEY COMPREHENSIVE PLAN 2020 Mid-Term Update. 2021. Available from: https://bouldercolorado.gov/media/3350/download?inline. Cited 2024 Apr 20

[121] Mintzer M, Mendoza J, Chawla L, Dellepiane A. Growing Up Boulder: Young People’s Ideas for 15-Minute Neighborhoods. 2016. Available from: https://www.colorado.edu/cedar/sites/default/files/attached-files/15_min_neighborhood_report.pdf. Cited 2024 Apr 20

[122] Macintyre S, Ellaway A, Cummins S. Place effects on health: how can we conceptualise, operationalise and measure them? Soc Sci Med. 2002;55(1):125–39. 12137182 10.1016/s0277-9536(01)00214-3

[123] Colls R, Evans B. Making space for fat bodies?: A critical account of ‘the obesogenic environment.’ Prog Hum Geogr. 2014;38(6):733–53.

[124] Andrews GJ, Hall E, Evans B, Colls R. Moving beyond walkability: on the potential of health geography. Soc Sci Med. 2012;75(11):1925–32. 22954467 10.1016/j.socscimed.2012.08.013

[125] Olsen JR, Nicholls N, Panter J, Burnett H, Tornow M, Mitchell R. Trends and inequalities in distance to and use of nearest natural space in the context of the 20-min neighbourhood: A 4-wave national repeat cross-sectional study, 2013 to 2019. Environ Res. 2022;213:113610. 35690087 10.1016/j.envres.2022.113610

[126] Elldér E. Built environment and the evolution of the “15-minute city”: A 25-year longitudinal study of 200 Swedish cities. Cities. 2024;149:104942.

[127] AlWaer H, Cooper I. Unpacking the concept of 20-minute neighbourhoods: disentangling “desired outcomes” from the “means” available for achieving them. Open House Int. 2023;48(4):704–28.

[128] Lamb KE, Daniel M, Chaix B, Kestens Y, Coffee NT, Thornton LE. Socioeconomic differences in associations between living in a 20-min neighbourhood and diet, physical activity and self-rated health: Cross-sectional findings from ProjectPLAN. Health Place. 2023;84:103119. 37742399 10.1016/j.healthplace.2023.103119

[129] British Academy, Academy of Medical Sciences. Historic and Geographic Patterns of Health Inequalities. London; 2022 p. 1–38. Available from: https://www.thebritishacademy.ac.uk/documents/3662/Historic_and_Geographic_Patterns_of_Health_Inequalities_-_report.PDF. Cited 2023 Dec 13

[130] Höhn A, Stokes J, Pollack R, Boyd J, Chueca Del Cerro C, Elsenbroich C, et al. Systems science methods in public health: what can they contribute to our understanding of and response to the cost-of-living crisis? J Epidemiol Community Health. 2023;77(9):610–6. 37328262 10.1136/jech-2023-220435 PMC10423532

