# Supplementary material for: How could 20-minute neighbourhoods impact health and health inequalities? A policy scoping review
Source: BMC Public Health. 2024 Dec 18;24:3426. doi: 10.1186/s12889-024-20928-5 (PMC11653922; doi:10.1186/s12889-024-20928-5)
Supplement: Supplementary file 1 — Supplementary Material 1. [file 12889_2024_20928_MOESM1_ESM.docx]

**Appendix 1**

SEARCH STRATEGIES

**Example: Ovid (Medline & Embase)**

Note: Deduplicated within Ovid platform prior to export. Preferring records with abstract and starting from Embase as primary data source.

1 “*minute city”.ab,ti.

2 “*minute neighbo*”.ab,ti.

3 “*minute communit*”.ab,ti.

4 “complete communit*”.ab,ti.

5 “walkable neighbo*”.ab,ti.

6 “liveable neighbo*”.ab,ti.

7 compact city.ab,ti.

8 active.ab,ti.

9 compact urban.ab,ti.

10 chrono-urban.ab,ti.

11 superblock.ab,ti.

12 isobenefit urban.ab,ti.

13 “neighbo* unit”.ab,ti.

14 1 or 2 or 3 or 4 or 5 or 6 or 7 or 8 or 9 or 10 or 11 or 12 or 13

15 limit 14 to yr=”1980-Current”

16 limit 15 to english language

17 remove duplicates from 16

**Appendix 2**

*Summary of main pathways by city (grouped health outcomes)*

| Place | Concept element | Mechanism | Health outcome (grouped) |
| --- | --- | --- | --- |
| Newham, Singapore, Brampton, Chicago, Melbourne, Western Australia, Tempe, Vancouver, Ottawa | active travel | increased physical activity/reduced obesity | Health, mental health, physical health, NCD |
| Milan, Edinburgh, Vancouver, Newham, Sydney, Auckland, Budapest, Greater Bendigo, Melbourne, Kirkland, | active travel | reduced car emissions/pollution | health, physical health, NCD, mental health |
| Milan, Sydney, Kirkland, Tempe | active travel | community liveability | communicable diseases, health |
| Leeds | active travel, increased service accessibility | community liveability, local economy/employment support | health |
| Barcelona, Hounslow | active travel, net zero | reduced car emissions/pollution | health |
| Brampton, Kirkland, Boulder, Portland | active travel | NA | NCD, health |
| Singapore, Greater Adelaide | active travel, Improved mobility | NA | NCD |
| Toronto, Scotland, Vancouver, Auckland | increased service accessibility | community liveability | Health, mental health, physical health |
| Bangkok, Tempe | increased service accessibility | increased physical activity/reduced obesity | NCD, health |
| Bangkok, Chicago, Tempe | increased service accessibility | accessible/healthy food | NCD, health |
| Shanghai, Brampton, Tempe | increased service accessibility | improved healthcare | health |
| Auckland, Melbourne, Hamilton Kirikiriroa, Tempe | increased service accessibility | NA | health |
| Toronto, Scotland, Budapest | increased service accessibility, nature access/green or blue infrastructure | NA | health |
| Melbourne, Tempe | increased service accessibility, nature access/green or blue infrastructure | increased physical activity/reduced obesity | Mental health, physical health |
| Vancouver | increased service accessibility, nature access/green or blue infrastructure | community liveability | health, physical health, mental health |
| Oslo, Singapore, Brampton | increased service accessibility, improved built environment | community liveability | health, mental health, physical health |
| Milan | increased service accessibility, improved built environment | sustainability | health |
| Greater Bendigo | increased service accessibility, improved built environment | increased physical activity/reduced obesity | NCD, health |
| Edinburgh | increased service accessibility, improved service quality | community liveability | health |
| Scotland | increased service accessibility, improved built environment | reduced car emissions/pollution | health |
| Chicago | Increased service accessibility, improved service quality | improved healthcare | mental health |
| Sydney | increased service accessibility, improved housing | community liveability | health |
| Sydney | increased service accessibility, net zero, nature access/green infrastructure | sustainability | health |
| Greater Adelaide | increased service accessibility, net zero | sustainability | Physical health, mental health |
| Oslo, Edinburgh | improved built environment | sustainability | health |
| Boulder | improved built environment | increased physical activity/reduced obesity | Health |
| Boulder | improved built environment |  | Health, mental health |
| Buenos Aires | improved built environment, improved mobility | community liveability | health |
| Auckland | nature access/green or blue infrastructure | NA | health |
| Auckland | Improved service quality | Improved healthcare | Mental health |
| Auckland, Tempe | Improved housing | NA | health |
| Budapest, Greater Bendigo, Auckland, Portland | NA | community liveability | Health, NCD, accidents |
| Central Puget Sound Region | NA | reduced car emissions/pollution | NCD |
| Auckland | NA | Improved healthcare | Communicable diseases |
| Melbourne | NA | increased physical activity/reduced obesity | NCD |
